# Management of acute mastoiditis in children: a retrospective analysis

**DOI:** 10.1186/s13052-025-02075-8

**Published:** 2025-09-24

**Authors:** Marella Reale, Carlotta Montagnani, Pietro Orlando, Luca Mazzetti, Marco Trinci, Luca Leone, Mariapaola Guidi, Giuseppe Indolfi, Sandra Trapani, Franco Trabalzini, Luisa Galli

**Affiliations:** 1 Otorhinolaryngology Unit, Head and Neck Department, Meyer Children’s Hospital IRCCS, Florence, Italy; 2 Infectious Diseases Unit, Department of Pediatrics, Meyer Children’s Hospital IRCCS, Florence, Italy; 3 Department of Otorhinolaryngology, Careggi University Hospital, Florence, Italy; 4 Department of Pediatrics, Meyer Children’s Hospital IRCCS, Florence, Italy; 5 Department Neurofarba, University of Florence, Florence, Italy; 6 Department of Health Sciences, University of Florence, Florence, Italy; 7 Department of Experimental and Clinical Medicine, University of Florence, Florence, Italy

**Keywords:** Acute mastoiditis, Complicated acute mastoiditis, Acute otitis media, Children

## Abstract

**Background:**

Acute mastoiditis (AM) is the most common complication of acute otitis media (AOM) and could lead to serious complications if not diagnosed early and treated appropriately. Nowadays, there is no definitive consensus about the diagnostic algorithm and the optimal therapeutic management for patients with AM. The purpose of this study is to analyze the management of children admitted for AM and complicated AM (CAM) in a referral children’s hospital, evaluating differences in clinical presentation and management to outline a diagnostic and therapeutic pathway. Moreover, the incidence over time was assessed.

**Methods:**

Retrospective study of children admitted for AM at Meyer University Hospital– IRCCS, Florence from January 2016 to December 2023.

**Results:**

Eighty-five patients were included in the study (60% male, median age 4 years), the microbiological examinations were carried out in 68% of them. The most frequent isolated pathogens were *Pseudomonas aeruginosa* in AM and *Streptococcus pyogenes* in CAM. Seventeen patients developed a CAM. An elevated CRP value is associated with an increased risk of CAM (*p* = 0.043). Management of patients with AM was mainly medical with intravenous antibiotics. Surgical intervention was required only in one case (1 out of 68). In contrast, surgical intervention was required in 76% of CAM cases (13 out of 17). The most common procedure was mastoidectomy combined to abscess drainage, according to the predominance of this complication in our study group. Only one patient had a recurrence leading to a second surgery. No significant statistical correlation was found between the occurrence of complications and younger age, personal history of otitis or leukocyte count. A significant increase in AM case was found during the study period.

**Conclusions:**

AM and CAM are infrequent but potentially life-threatening complications of AOM. A marked rise in AM cases was observed in 2023, likely due to the lifting of pandemic restrictions. A heterogeneous management of mastoiditis was observed, even within a single center. Elevated CRP levels are the only identified parameter associated with the complicated form. Pediatricians should be aware of the importance of a prompt diagnosis and guidelines should be developed to support effective management.

**Supplementary Information:**

The online version contains supplementary material available at 10.1186/s13052-025-02075-8.

## Background

Acute mastoiditis (AM) is the most common complication of acute otitis media (AOM), occurring in 1/400 patients [1], but it remains a relatively rare condition overall. Its incidence varies worldwide and reaches 1.2–6.1/100.000 cases/year in children aged 0–14, due to their favorable anatomy, immunological immaturity, and recurrent airway infections [2, 3]. Although the incidence and severity of acute mastoiditis (AM) have decreased with the introduction of vaccines and antibiotics, it remains a potentially life-threatening condition due to the risk of developing complicated AM (CAM). Common complications include mastoid and intracranial abscesses, cranial nerve (CN) palsies, meningitis, adjacent bone osteomyelitis, and cerebral venous sinus thrombosis (CVST) [4–7]. Currently, there are no universally accepted guidelines for the management of AM and CAM, resulting in significant variability in clinical practice. There is no consensus on the role of imaging, choice of antibiotic therapy and surgery indications [8]. The diagnosis of AM is mainly clinical, based on retroauricular swelling, erythema, and tenderness, often accompanied by auricle protrusion and, in some cases, otorrhea, typically in a child with a history of AOM [4, 9, 10]. Paradoxically the signs and symptoms of CAM may be subtle, leading to delayed diagnosis and an increased risk of long-term neurological sequelae (e.g. hemiparesis, hydrocephalus, mental retardation, polyneuropathy, epilepsy) and mortality [11, 12]. This study aimed to define a practical diagnostic and therapeutic approach for AM and CAM by retrospectively reviewing electronic medical records of patients treated at the pediatric department of a children’s referral hospital. We specifically analyzed clinical presentation, laboratory finding at admission, microbiological results, antibiotics regimens, surgical interventions, and outcomes, comparing the two patient groups.

## Methods

We conducted a retrospective, single-centre study including all patients admitted to Meyer Children’s Hospital IRCCS, Florence, Italy, for AM and CAM, from January 1st, 2016, to December 31st, 2023. The diagnosis of AM was clinical and based on the presence of swelling, erythema, and tenderness of the retroauricular region, or the protrusion of the auricle in a child with concomitant AOM or a history of AOM in the past 4 weeks, with or without consistent radiological findings. The diagnosis of CAM was established in the case of clinical suspicion and radiological confirmation of meningitis, osteomyelitis, intracranial and mastoid abscess, CVST, and/or VI and VII CN palsy.

Exclusion criteria were comorbid cholesteatoma, patients with cochlear implants and neurological signs with no involvement of mastoid cells.

Electronic medical records were retrospectively reviewed. Demographic, anamnestic, and clinical data were collected: sex, age, comorbidities, prior antibiotic therapy, white blood count (WBC), neutrophil count, C-reactive protein levels (CRP), pathogens at the microbiological examinations, radiological findings (e.g. meningeal and/or bone enhancement, the presence of mastoid and/or intracranial abscess, the absence of flow into a cerebral sinus, enhancement along the course of a cranial nerve) at computed tomography (CT) scan or Magnetic Resonance Imaging (MRI), antibiotics and other drugs administration during hospitalization, surgical approach, postoperative complications, length of hospital stay, and further admission to the otorhinolaryngology department when available. Bacteria from drained purulent material were identified by cultural or molecular methods (Polymerase Chain Reaction, PCR).

Results were reported according to the Strengthening the Reporting of Observational studies in Epidemiology (STROBE) guidelines [13].

Patients were categorized into two groups, AM and CAM, and data were compared.

At our institution a diagnostic-therapeutic protocol has not been developed yet.

### Statistical analysis

The statistical analyses were performed by SPSS software (IBM Corp. Released 2020. IBM SPSS Statistics for Macintosh, Version 27.0. Armonk, NY: IBM Corp). Continuous variables were expressed as mean ± standard deviations or median and interquartile ranges (IQR), as appropriate. The Shapiro-Wilk test was used to check for the normality of data. The unpaired two-tailed Student’s t-test and the Mann-Whitney test (for normally and non-normally distributed variables, respectively) were used to compare the mean scores for each item between subgroups. Categorical variables were presented as counts and percentages. A logistic regression model was then built for the outcome “complication”, including clinically relevant variables as covariates. The goodness of fit of the model was assessed using the Hosmer-Lemeshow test. Possible final models were evaluated comparing their respective Akaike Information Criterion and Bayesian Information Criterion where indicated. Intraclass correlation was then calculated. The optimal cutoff value for CRP levels was determined using Receiver Operating Characteristic curve analysis. The sensitivity and specificity were calculated for each possible threshold, and the optimal cutoff was identified as the value that maximized the Youden Index (J = Sensitivity + Specificity − 1). All statistical tests were two-sided and a p-value of less than 0.05 was considered statistically significant.

## Results

Overall, 90 patients were admitted to our hospital with a diagnosis of AM in the study period. However, 5 of them were excluded, 3 because of comorbid cholesteatoma, and 2 because of facial palsy from AOM without mastoid involvement. Hence, 85 patients were included, of whom 68 were affected by AM (80.0%) and 17 by CAM (20.0%).

Patient characteristics are summarized in Table 1. No statistically significant differences were found with regard to sex, age, side, and history of previous otitis or mastoiditis. Considering the general comorbidities, the most frequent ones were neurological or syndromic diseases, while adenotonsillar hypertrophy was the most common ENT (ear, nose, and throat)-related comorbidity.

**Table 1:** Demographic, clinical, laboratory and treatment characteristics of the study population page 4, line 116

	Total (85)	AM (68)	CAM (17)	*p*
**General characteristics**				
Male n (%)	52 (61.2%)	41 (60.3%)	11 (64.7%)	0.738
Age months (median; IQR)	51.8; 27.7–94.6	60.8; 24–100	44; 28.9–90	0.303
Previous AOM n (%)	27 (31.8%)	20 (29.4%)	7 (41.2%)	0.522
Previous AM n (%)	2 (2.4%)	2 (2.9%)	0	0.474
Comorbidities n (%)	8 (9.4%)	7 (10.3%)	1 (5.9%)	0.579
ORL Comorbidities (%)	10 (11.8%)	8 (11.8)	2 (11.8%)	> 0.999
**Clinical and laboratory characteristics**				
Right side n (%)	46 (54.1%)	38 (55.9%)	8 (47.1%)	0.703
Bilateral n (%)	3 (3.5%)	2 (2.9%)	1 (5.9%)	0.985
Swelling, erythema, and tenderness of the RA n (%)	66 (77.6%)	57 (83.8%)	9 (52.9%)	**0.016**
Protrusion of the auricle n (%)	57 (67.1%)	49 (72.1%)	8 (47.1%)	**0.05**
TM inflammation n (%)	51 (60%)	42 (61.8%)	9 (52.9%)	0.698
TM perforation n (%)	17 (20%)	13 (19.1%)	4 (23.5%)	0.946
Endotympanic effusion n (%)	25 (29.4%)	19 (27.9%)	6 (35.3%)	0.766
Otorrhea n (%)	38 (42.2%)	30 (44.1%)	8 (33.3%)	0.869
Fever n (%)	36 (42.4%)	32 (47%)	4 (23.5%)	0.134
Lymphadenopathy n (%)	28 (32.9%)	24 (35.3%)	4 (23.5%)	0.356
VI CN Palsy n (%)	2 (2.3%)	0	2 (11.8%)	**0.007**
VII CN Palsy n (%)	4 (4.4%)	0	4 (18.2%)	**0.001**
Vomiting n (%)	8 (9.4%)	6 (8.8%)	2 (11.8%)	0.658
WBC count (*1000/mcL) mean (SD)	15.63 (6.45)	15.42 (6.16)	16.31 (7.75)	0.551
Neutrophils count (1000/mcL) Media (SD)	10.8 (6.15)	10.77 (5.75)	11.08 (7.85)	0.472
CRP (mg/dL) mean(SD)	9.21 (8.44)	8.44 (7.85)	11.82 (10.25)	**0.043**
**Treatment characteristics**				
Antibiotic therapy at home n (%)	45 (52.9%)	33 (48.5%)	12 (70.6%)	0.052
Days of antibiotic therapy at home mena (SD)	4.5 (3.4)	4.4 (3.5)	4.7 (3.1)	0.760
Steroid therapy at home n (%)	6 (7.2%)	5 (7.6%)	1 (5.9%)	0.740
NSAIDs therapy at home n (%)	25 (38.5%)* *20 missing	19 (37.2%)* *17 missing	6 (42.8%)* *3 missing	0.801
Initial AB monotherapy (n, %)	56 (65.9)	50 (73.5%)	6 (35.3%)	**0.007**
Final combined AB regimen (n, %)	67 (78.8%)	51 (75%)	16 (94.1%)	0.140
Length of AB treatment ( mean, SD)	20.36 (7.8)	18.32 (5.3)	28.5 (10,7)	**< 0.001**
Steroids treatment (n, %)	14 (16.5%)	5 (7.3)	9 (52.9)	**< 0.001**
Surgery	14 (16.5%)	1 (1.5%)	13 (76.5)	**< 0.001**

Legend. AM: acute mastoiditis; CAM: complicated AM; IQR: interquartile range; AOM: acute otitis media; NSAIDs: nonsteroidal anti-inflammatory drugs; RA: retro-auricular area; CN: cranial nerve; AB: antibiotic; SD: standard deviation

Most patients sought medical attention due to swelling, erythema, and tenderness of the retroauricular area (77,6%). The aforementioned clinical signs were significantly more frequent in patients without complications (*p* = 0.016). In contrast, VI and VII CN palsies were characteristic features of patients diagnosed with CAM (*p* = 0.007 and *p* = 0.001, respectively). Regarding blood tests, only CRP levels were significantly higher in patients with CAM (*p* = 0.043), while no significant differences were observed in WBC or neutrophil counts.

According to logistic regression analysis, an increased risk of CAM was observed in patients with increased CRP levels [Odds Ratio (OR) 1.082, 95%CI: 1.008–1.162, *p* = 0.029] whereas a reduced risk was observed in patients presenting with swelling, erythema, and tenderness of the retroauricular area (OR 0.173, 95%CI: 0.031–0.976, *p* = 0.047). Due to the scarcity of events for VI and VII CN palsies (2 and 4, respectively), the logistic regression model failed to produce reliable estimates for these variables, leading to convergence issues and unstable coefficient values. As a result, their predictive value could not be accurately assessed.

The optimal cutoff value identified for CRP was 12 mg/dL, above which the risk of CAM significantly increased.

Regarding diagnostic imaging, 33 out of 85 patients (38.8%) underwent a CT scan (26.5% in AM group vs. 88.2% in CAM group, *p* < 0.001) and 14 patients (16.5%) underwent a MRI (8.8% in AM group vs. 47.1% in CAM group, *p* < 0.001).

About half of patients (45/85, 52.9%) received empiric antibiotic therapy at home before hospital admission, with a mean length of 4.7 days (p 0.051). In detail, 27 patients (31.8%) were treated with amoxicillin/clavulanic acid, 11 (12.9%) with third-generation cephalosporins, and 3 (3.5%) with amoxicillin, with no significant differences between AM and CAM groups. Unfortunately, data on doses were not available. No difference was observed in Non-steroidal anti-inflammatory drugs (NSAIDs) administration, although in a non-negligible proportion of cases (23.5%) data regarding home administration of NSAIDS were missing.

**Fig. 1:**
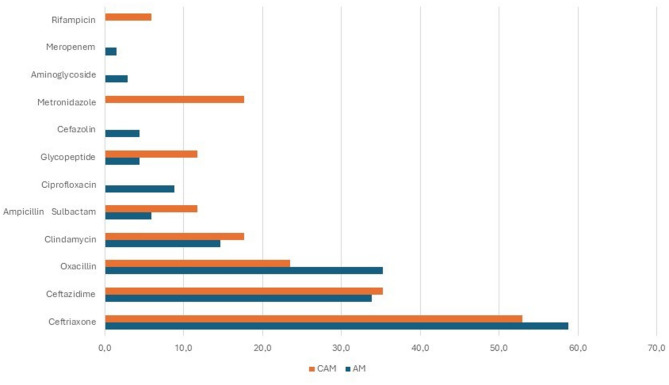
Antibiotic treatment (%). Legend. AM: acute mastoiditis; CAM: complicated AM

After admission, 84/85 patients received intravenous antibiotic therapy (IVAT). Initial monotherapy was more frequent in the AM group (73.5% vs. 35.3%, p 0.007). Initial antibiotic treatment was subsequently modified in 51 patients (60.0%), based on culture results or due to inadequate clinical response. Overall, 18 different antibiotic regimens were used, with ceftriaxone being the most commonly drug administered in both groups. Treatment schedules are shown in Fig. 1.

The mean duration of IVAT therapy was significantly longer in the CAM group than the AM group (28.5 ± 10.7 days vs. 18.32 ± 5.3 days, *p* < 0.001), as well as the mean length of hospital stay (9.1 ± 3.1 days vs. 14.5 ± 4.8 days, *p* < 0.001). Only 14 patients (16.5%) received concomitant intravenous steroid therapy, 5 in the AM and 9 in the CAM group (*p* < 0.001) and anticoagulant treatment was administered to patients with CVST with a mean duration of 96.2 ± 22.7 days.

**Fig. 2:**
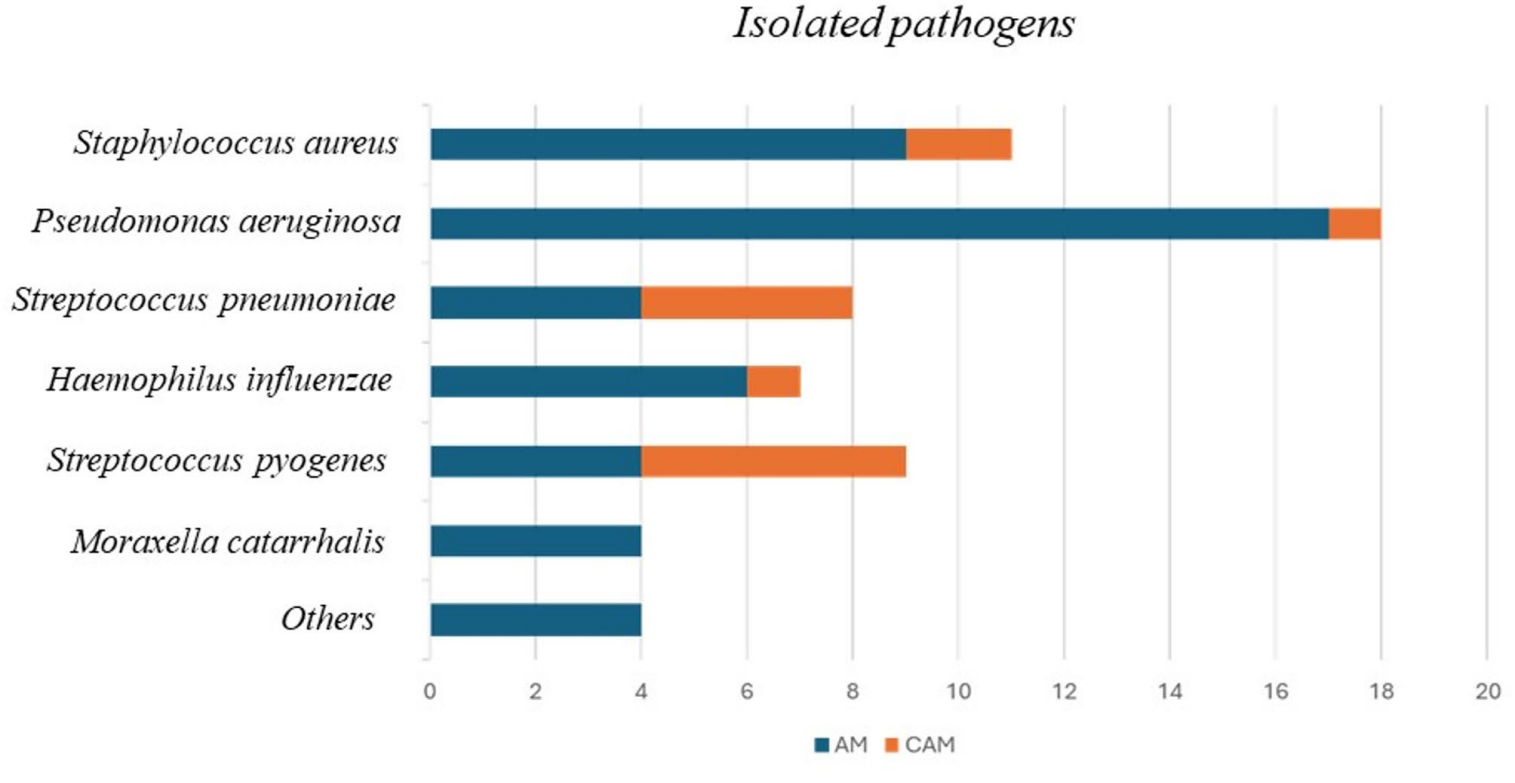
Pathogens isolated through molecular and cultural examinations and number of patients. Legend. AM: acute mastoiditis; CAM: complicated AM

Microbiological investigations by means of molecular and/or culture methods were performed on 31 patients (26 AM and 5 CAM) using otorrhea samples and on 10 CAM patients who underwent surgery using drained material. The most commonly isolated pathogen in the AM group was *Pseudomonas aeruginosa*, followed *by Staphylococcus aureus* and *Haemophilus influenzae*. In contrast, the most frequently isolated pathogen in the CAM group was *Streptococcus pyogenes*, followed by *Streptococcus pneumoniae*. Microbiological findings are reported in Fig. 2.

Overall, 14 patients required surgery, including 13 in the CAM group (76.5%) and 1 in the AM group (1.5%, *p* < 0.001), due to a lack of clinical improvement despite IVAT. Indications for surgery included mastoid abscesses (7, 41.2%) one of them associated with meningitis, sinus thrombosis (5, 29.4%) one of them associated with meningitis and one with VI cranial nerve palsy, epidural abscess (1, 5.9%), VII CN palsy (1, 5.9%). However, 3 patients with VII cranial nerve palsy and 1 patient with VI cranial nerve palsy achieved full nerve recovery with medical therapy alone. Surgical approaches varied depending on the type of complication (Table 2).

**Table 2:** Surgical procedures. Page 6, line 166

Type of surgical procedure	Surgical Patients (14)	AM (1)	CAM (13)	Clinical condition requiring surgery
**Mastoidectomy with abscess drainage n (%)**	7 (50%)	0	7 (53.8%)	**7 mastoid abscesses**
**Mastoidectomy n (%)**	6 (28.6%)	1 (100%)	5 (23.1%)	**5 venous thrombosis and 1 chronicity**
**Abscess drainage without mastoidectomy n (%)**	1 (7.1%)	0	1 (7.7%)	**1 epidural abscess**

Legend. AM: acute mastoiditis; CAM: complicated AM

Mastoidectomy with abscess surgical drainage was the most common procedure. No postoperative complications were reported but one patient with sinus thrombosis developed intracranial hypertension that was successfully treated with corticosteroids and acetazolamide. Unfortunately, one patient needed a second mastoid surgery for a new episode of CAM that occurred one month later, while another patient needed a myringotomy for a new episode of AOM that arose 10 days after home discharge.

**Fig. 3:**
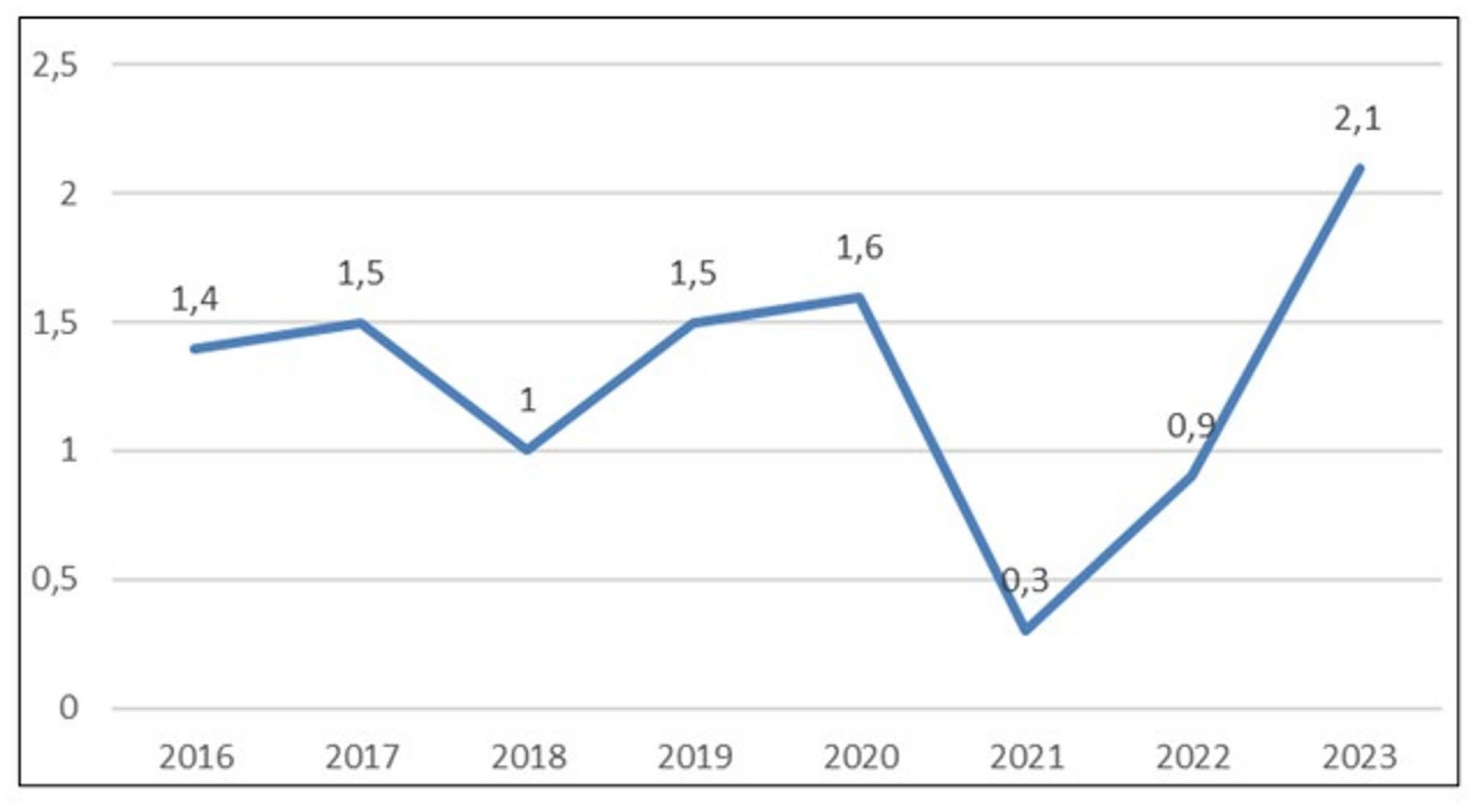
Case of acute mastoiditis/1000 discharged patients

The incidence of AM per 1,000 discharged patients was analyzed throughout the study period. A significant decrease in incidence was observed during the SARS-CoV-2 pandemic period (2020–2022), followed by a statistically significant peak in 2023 (*p* = 0.01), as shown in Fig. 3.

## Discussion

AM and CAM were uncommon but severe complications of AOM, particularly in children. To date, no universally accepted guidelines are available about the appropriate diagnostic and therapeutic workout [8, 14]. Thus, the management of AM or CAM may consistently depend on the available professional specialties in a certain hospital and on the physicians’ experience.

In this context, our study aimed to share our experience at an Italian third-level pediatric university hospital in managing AM and CAM. Secondly, we aimed to compare our findings with those reported by the current literature, as the incidence of AM and CAM have been increasing after the SARS-CoV-2 pandemic period [15, 16].Notably, we found a higher median age at admission than the literature reported. Several studies reported a median age of 2 years [2, 3, 13, 14], whereas we found a median age of about 4 years. This finding may be explained by the enactment of the Italian guidelines provided in 2019 recommending immediate antibiotic therapy in children < 2 years old diagnosed with AOM and a wait-and-see strategy for older ones [17].

Consistently to the current literature, the diagnosis of AM was essentially driven by signs and symptoms, in particular, the presence of swelling, erythema, and tenderness of the retroauricular region with or without the protrusion of the auricular pinna was robustly associated with an AM diagnosis [8, 18]. In contrast, CAM diagnosis may be challenging when considering clinical features alone, as most patients were initially admitted for neurological disorders [6]. Hence, an otological origin should not be underestimated in a child complaining of a neurological disorder and a history of previous AOM. In such a similar scenario, a CT scan or an MRI were mandatory. The statistically higher number of CT scans in the CAM group may be explained by the radiation-sparing policy of our hospital, where a CT scan was performed only in cases of neurological signs or missed clinical improvement after a 48-hour IVAT schedule. In addition, we found a significantly increased CRP in patients with CAM, and higher but not significant WBC and neutrophils count. Thus, an elevated CRP value (in our cohort, above 12 mg/dL) at presentation should prompt the clinician to suspect a complicated form of the disease. This finding is consistent with literature from other countries (e.g. United Kingdom, Spain, Israel) although in these studies the higher value of CRP was not significant [19–22].

On the whole, 17 out of 85 patients were finally diagnosed with CAM (20.0%) at our center throughout the study period, a value that is in line with existing literature [17, 18]. Although our sample size was limited, the incidence of AM and CAM was similar to other studies over the SARS-CoV-2 pandemic and post-pandemic period [19, 20]. Many recent studies have reported an increase in AM and its complications after COVID-19 restrictions were lifted [15, 16]. In our study, we noticed a decreased incidence during the SARS-CoV-2 pandemic period, likely due to the containment measures and social distancing, while a significant increase in the post-pandemic period was observed, consistently with other respiratory infections in children [15, 23–25].

The high rate of CAM in our cohort may be partially attributed to the referral nature of our center, where difficult-to-treat patients from other facilities are concentrated. Indeed 11/85 patients had prior hospitalization in a non-tertiary center where a pediatric-specific multidisciplinary team was not available. The lack of a dedicated team may have contributed to a delayed diagnosis and the increased risk of developing a CAM. Furthermore, patients with CAM had a higher rate of previous home empiric antibiotic therapy, nonetheless, they did not have a shorter hospital stay compared to the AM group as reported by other studies [26]. Probably, they were patients with the worst baseline condition so their general doctors prescribed more antibiotics, however, we cannot establish whether a wrong antibiotic regimen was a risk factor for complications development. Notably, among patients for whom information on home treatment was available, the most frequently used antibiotic was amoxicillin-clavulanic acid, which, although broader in spectrum, does not align with current European guidelines for AOM that recommend amoxicillin monotherapy [27]. Nevertheless, the use of amoxicillin-clavulanic acid may be a more effective empirical choice in certain clinical settings, given the high rate of β-lactamase production by gram-negative microorganisms such as *H. influenzae* and *Moraxella catarrhalis*.

*P. aeruginosa* was the most frequently isolated bacteria in the AM group and *S. pyogenes* in the CAM group. Historically, *S. pneumoniae*,* S. pyogenes*, and *H. influenzae* have historically been recognized as the most common causes of AM and CAM. The pathogenic role of *P. aeruginosa*, however, remains uncertain. Several authors suggest that *P. aeruginosa* should be considered a contaminant originating from the outer ear, and that this pathogen causes a less aggressive form of disease [2, 28–31]. This hypothesis is supported by the fact that *P. aeruginosas* was rarely isolated in complicated cases within our cohort.

At admission, almost all patients with AM started broad-spectrum IVAT with third-generation cephalosporins or ampicillin/sulbactam to cope with *S. pneumoniae*, *S. pyogenes*, and *H. influenzae* [32]. IVAT was later modified according to the molecular or cultural findings on otorrhea material in over 60% of patients. Eighteen different schedules were employed, and this may reflect the absence of local and international guidelines. Indeed, therapeutic choices were based on the individual judgment of the attending clinicians rather than on a predefined protocol, which explains the observed variability in treatment approaches. According to the current literature, in our cohort third-generation cephalosporins (ceftriaxone or ceftazidime) were the most commonly used antibiotics, alone or in combination with another antibiotic [33–35]. Unfortunately, due to the small sample size and the retrospective nature of the study, it is not possible to determine whether a specific therapeutic regimen was initiated in response to the development of a complication or may have contributed to it. As a result, no definitive conclusions can be drawn regarding the relative effectiveness of different treatment strategies. Most patients fully recovered with IVAT alone, so it appeared to be the most appropriate first-line strategy in patients with AM as per the current evidence. However, more studies are needed to discern whether the third-generation cephalosporins should be used alone or in combination, and which one is the most appropriate combination. Moreover, multidisciplinary management (e.g. pediatrician, ENT, and infectivologist) should be also considered as an effective tool for an early diagnosis and a correct therapeutic approach.

In the case of CAM, surgery was the mainstay of treatment, followed by IVAT. Patients underwent mastoidectomy associated with abscess drainage in the event of abscess’ presence. We did not report postoperative complications and only one patient needed an abscess drainage one month after the first surgery for a new episode of AM. A recently published review stated mastoidectomy to be the most effective treatment option in patients affected with CAM, so we may speculate that mastoidectomy should represent the treatment of choice in patients with CAM [8, 36].

Certainly, the low number of patients and the short follow-up period represented the major limitations of our study, as well as its retrospective nature which may have affected the accuracy of diagnosis due to possible deficiencies in clinical records and variability in imaging findings. We recognize that further studies on larger cohorts and longer follow-up periods are warranted to highlight possible signs, symptoms, laboratory, and radiological findings that may facilitate the diagnosis of CAM. Expert agreement and recommendations about the most appropriate diagnostic and treatment work-up are needed to cope with these two rare but severe entities.

## Conclusion

AM and CAM are infrequent but potentially life-threatening complications of AOM, a very common disease among children. A significant increase in AM cases has been reported after the SARS-CoV-2 pandemic. Nonetheless, universally accepted consensus statements are still lacking, and management of affected patients is quite heterogeneous among different hospitals. Clinical signs, as well as laboratory and radiological findings, are fundamental for the early detection of AM-related complications. CAM should always be considered in the differential diagnosis in the case of neurological signs in a child with a history of AOM. Further, prompt IVAT and adequate surgery are mandatory in the treatment of AM and CAM, respectively.

## Electronic supplementary material

Below is the link to the electronic supplementary material.

Supplementary Material 1

Supplementary Material 2

## Data Availability

The datasets used and/or analysed during the current study are available from the corresponding author on reasonable request.

## Abbreviations

AM: Acute mastoiditis
AOM: Acute otitis media
CAM: Complicated acute mastoiditis
CN: Cranial nerve
CRP: C-reactive protein
CT: Computed tomography
CVST: Cerebral venous sinus thrombosis
ENT: Ear, nose, and throat
IQR: Interquartile ranges
IVAT: Intravenous antibiotic therapy
MRI: Magnetic Resonance Imaging
NSAIDs: Non-steroidal anti-inflammatory drugs
OR: Odds ratio
PCR: Polymerase Chain Reaction
STROBE: Strengthening the Reporting of Observational studies in Epidemiology
WBC: White blood count
