# Supplementary material for: Management of acute mastoiditis in children: a retrospective analysis
Source: Ital J Pediatr. 2025 Sep 24;51:272. doi: 10.1186/s13052-025-02075-8 (PMC12462347; doi:10.1186/s13052-025-02075-8)
Supplement: Supplementary file 1 — Supplementary Material 1 [file 13052_2025_2075_MOESM1_ESM.pdf]

Change of authorship request form - Journals (pre-acceptance)

Important information. Please read.

- This form should be used by authors to request any change in authorship (adding/deleting authors) including changes in corresponding authors. This form should not be used for name changes. Please fully complete all sections. Use black ink and block capitals and provide each author's full name with the given name first followed by the family name.

- By signing this declaration, all authors guarantee that the order of the authors are in accordance with their scientific contribution, if applicable as different conventions apply per discipline, and that only authors have been added who made a meaningful contribution to the work.

- Please note, in author collaborations where there is formal agreement for representing the collaboration, it is sufficient for the representative or legal guarantor (usually the corresponding author) to complete and sign the Authorship Change Form on behalf of all authors, **next to the added/removed author(s). (Complete Section 3, followed by Section 6.)**
  In author collaborations where there is no formal agreement for representing the collaboration and **there are more than 10 authors**, one may sign for all, provided the signer appends correspondence that attests that each of the authors have agreed to the change **and the added/removed authors sign the form. (Complete Section 3, followed by Section 6.)**

- Please note, we cannot investigate or mediate any authorship disputes. If you are unable to obtain agreement from all authors (including those who you wish to be removed) you must refer the matter to your institution(s) for investigation. Please inform us if you need to do this.

- If you are not able to return a fully completed form within **30 days** of the date that it was sent to the author requesting the change, we may have to withdraw your manuscript. We cannot publish manuscripts where authorship has not been agreed by all authors (including those who have been removed).

- Incomplete forms will be rejected.

- Please return/upload this form, fully completed, to the Journals Editorial Office. The Journal and/or Publisher will consider the information you have provided to decide whether to approve the proposed change in authorship. We may decide to contact your institution for more information or undertake a further investigation, if appropriate, before making a final decision.

SPRINGER NATURE

Change of authorship request form - Journals (pre-acceptance)

Section 1: Please provide the current title of manuscript

Manuscript ID no.: ITJP-D-25-00518R1

Title: Management of acute mastoiditis in children: a retrospective analysis

Section 2: Please provide the previous authorship, in the order shown on the manuscript before the changes were introduced. Please indicate the corresponding author by adding (CA) behind the name.

	First name(s)	Family name	ORCID or SCOPUS id, if available
1st author	MARELLA	REALE	57204646767
2nd author	CARLOTTA (CA)	MONTAGNANI	0000-0001-5950-4965
3rd author	PIETRO	ORLANDO	0000-0002-2372-2407
4th author	LUCA	MAZZETTI	
5th author	MARCO	TRINCI	
6th author	LUCA	LEONE	
7th author	GIUSEPPE	INDOLFI	0000-0003-3830-9823
8th author	SANDRA	TRAPANI	7003335486
9th author	FRANCO	TRABALZINI	0000-0003-3207-1535
10th author	LUISA	GALLI	0000-0002-7912-3366

Please use an additional sheet if there are more than 10 authors.

SPRINGER NATURE Springer Nature is one of the world's leading global research, educational and professional publishers, created in May 2015 through the combination of Nature Publishing Group, Palgrave Macmillan, Macmillan Education and Springer Science+Business Media.

Change of authorship request form - Journals (pre-acceptance)

Section 3: Please provide a justification for change. Please use this section to explain your reasons for changing the authorship of your manuscript, e.g. what necessitated the change in authorship? Please refer to the (journal) policy pages for more information about authorship. Please explain why omitted authors were not originally included and/or why authors were removed on the submitted manuscript.

THE AUTHOR GAVE HELP IN THE REVIEWING PROCESS AND FOUND ALL REFERENCES WHICH WAS ADDED TO THE PAPER, HELPED THE AUTHORS TO MAKE THE IMPROVEMENT REQUESTED.

Section 4: Proposed new authorship. Please provide your new authorship list in the order you would like it to appear on the manuscript. Please indicate the corresponding author by adding (CA) behind the name. <u>If the Corresponding Author has changed, please indicate the reason under section 3</u>.

	First name(s)	Family name (this name will appear in full on the final publication and will be searchable in various abstract and indexing databases)	Affiliated institute	E-mail address
1st author	MARELLA	REALE	AOU MEYER	MARELLA.REALE@MEYER.IT
2nd author	CARLOTTA (CA)	MONTAGNANI	AOU MEYER	CARLOTTA.MONTAGNANI@MEYER.IT
3rd author	PIETRO	ORLANDO	AOU CAREGGI	PLUNPIR91@GMAIL.COM
4th author	LUCA	MAZZETTI	AOU MEYER	LUCA.MAZZETTI@UNIFI.IT
5th author	MARCO	TRINCI	AOU CAREGGI	MARCO.TRINCI@UNIFI.IT
6th author	LUCA	LEONE	AOU MEYER	LUCA.LEONE@MEYER.IT
7th author	MARIAPAOLA	GUIDI	AOU MEYER	MARIAPAOLA.GUIDI@MEYER.IT
8th author	GIUSEPPE	INDOLFI	AOU MEYER	GIUSEPPE.INDOLFI@MEYER.IT
9th author	SANDRA	TRAPANI	AOU MEYER	SANDRA.TRAPANI@MEYER.IT
10th author	FRANCO	TRABALZINI	AOU MEYER	FRANCO.TRABALZINI@MEYER.IT

Please use an additional sheet if there are more than 10 authors.

~~LUISA~~ ~~GALLI~~ ~~AOU MEYER~~ ~~LUISA.GALLI@MEYER~~.IT

SEE PAGE 2

SPRINGER NATURE Springer Nature is one of the world's leading global research, educational and professional publishers, created in May 2015 through the combination of Nature Publishing Group, Palgrave Macmillan, Macmillan Education and Springer Science+Business Media.

*

Change of authorship request form - Journals (pre-acceptance)

Section 3: Please provide a justification for change. Please use this section to explain your reasons for changing the authorship of your manuscript, e.g. what necessitated the change in authorship? Please refer to the (journal) policy pages for more information about authorship. Please explain why omitted authors were not originally included and/or why authors were removed on the submitted manuscript.

* PAGE 2

Section 4: Proposed new authorship. Please provide your new authorship list in the order you would like it to appear on the manuscript. Please indicate the corresponding author by adding (CA) behind the name. If the Corresponding Author has changed, please indicate the reason under section 3.

	First name(s)	Family name (this name will appear in full on the final publication and will be searchable in various abstract and indexing databases)	Affiliated institute	E-mail address
1 1st author	LUISA	GALLI	AOU MEYER	LUISA.GALLI@UNIFI.IT
2nd author				
3rd author				
4th author				
5th author				
6th author				
7th author				
8th author				
9th author				
10th author				

Please use an additional sheet if there are more than 10 authors.

SPRINGER NATURE Change of authorship request form - Journals (pre-acceptance)

Section 5: Author contribution, Acknowledgement and Disclosures. Please use this section to provide a new disclosure statement and, if appropriate, acknowledge any contributors who have been removed as authors and ensure you state what contribution any new authors made (if applicable per the journal or book (series) policy). Please ensure these are updated in your manuscript - after approval of the change(s) - as our production department will not transfer the information in this form to your manuscript.

New acknowledgements:

/

New Disclosures (financial and non-financial interests, funding):

This work was supported, in part, by funds from the 'Current Research Annual Funding' of the Italian Ministry of Health.

New Author Contributions statement (if applicable per the journal policy):

MG: interpretation of data, revised the work; approved the submitted version

State 'Not applicable' if there are no new authors.

SPRINGER NATURE Springer Nature is one of the world's leading global research, educational and professional publishers, created in May 2015 through the combination of Nature Publishing Group, Palgrave Macmillan, Macmillan Education and Springer Science+Business Media.

Change of authorship request form - Journals (pre-acceptance)

Section 6: Declaration of agreement. *All* authors, unchanged, new and removed *must* sign this declaration.

(NB: Please print the form, (docu)-sign and return/upload a scanned copy. Please note that signatures that have been inserted as an image file are acceptable as long as it is handwritten. Typed names in the signature box are unacceptable.) *** Please delete as appropriate. Delete all of the bold if you were on the original authorship list and are remaining as an author.**

	First name	Family name		Signature	Date
1st author	MARELLA	REALE	I agree to the proposed new authorship shown in section 4 ~~/and the addition/removal*of my name to the authorship list /and the proposed change in corresponding author~~	[illegible]	11 JUN 25
2nd author	CARLOTTA	MONTAGNANI	I agree to the proposed new authorship shown in section 4 /~~and the addition/removal*of my name to the authorship list /and the proposed change in corresponding author~~	[illegible]	11 JUN 25
3rd author	PIETRO	ORLANDO	I agree to the proposed new authorship shown in section 4 /~~and the addition/removal*of my~~ name to the authorship list ~~/and the proposed change in corresponding author~~	[illegible]	12 JUN 25
4th authors	LUCA	MAZZETTI	I agree to the proposed new authorship shown in section 4 /~~and the addition/removal*of my name to the authorship list /and the proposed change in corresponding author~~	[illegible]	12 JUN 25
5th author	MARCO	TRINGI	I agree to the proposed new authorship shown in section 4 ~~/and the addition/removal*of my name to the authorship list /and the proposed~~ change in corresponding author	[illegible]	12 JUN 25
6th author	LUCA	LEONE	I agree to the proposed new authorship shown in section 4 /~~and the addition/removal*of my name to the authorship list /and the proposed change in corresponding author~~	Luca Leone	12 JUN 25
7th author	MARIAPAOLA	GUIDI	I agree to the proposed new authorship shown in section 4 /and the addition/removal*of my name to the authorship list ~~/and the proposed change in corresponding author~~	Mariapaola Guidi	12 JUN 25

SPRINGER NATURE

Change of authorship request form - Journals (pre-acceptance)

	First name	Family name		Signature	Date
8th author	GIUSEPPE	INDOLFI	I agree to the proposed new authorship shown in section 4 /~~and the addition/removal*of my name to the authorship list /and the proposed change in corresponding author~~		12 JUN 25
9th author	SANDRA	TRAPANI	I agree to the proposed new authorship shown in section 4 /~~and the addition/removal*of my name to the authorship list /and the proposed change in corresponding author~~		12 JUN 25
10th author	FRANCO	TRABALZINI	I agree to the proposed new authorship shown in section 4 /~~and the addition/removal*of my name to the authorship list /and the proposed change in corresponding author~~		12 JUN 25

Please use an additional sheet if there are more than 10 authors.

~~LUISA~~ ~~GALLI~~ SEE SECOND PAGE* 12 JUN 25

In case of author collaborations with formal agreement:

	Name of consortium/consortia	First name	Family name		Signature	Date
Representative/legal guarantor				I agree to the proposed new authorship shown in section 4 /**and the addition/removal*of my name to the authorship list** /and the proposed change in corresponding author		

Both added/removed authors should complete the information in the first table under Section 6.

---- End of form ----

SPRINGER NATURE Springer Nature is one of the world's leading global research, educational and professional publishers, created in May 2015 through the combination of Nature Publishing Group, Palgrave Macmillan, Macmillan Education and Springer Science+Business Media.

SPRINGER NATURE

Change of authorship request form - Journals (pre-acceptance)

* PAGE 2

	First name	Family name		Signature	Date
11 8th author	LUISA	GALLI	I agree to the proposed new authorship shown in section 4 **/and the addition/removal*of my name to the authorship list** /and the proposed change in corresponding author	[illegible]	18JUN25
9th author			I agree to the proposed new authorship shown in section 4 **/and the addition/removal*of my name to the authorship list** /and the proposed change in corresponding author		
10th author			I agree to the proposed new authorship shown in section 4 **/and the addition/removal*of my name to the authorship list** /and the proposed change in corresponding author		

Please use an additional sheet if there are more than 10 authors.

In case of author collaborations with formal agreement:

	Name of consortium/consortia	First name	Family name		Signature	Date
Representative/legal guarantor				I agree to the proposed new authorship shown in section 4 **/and the addition/removal*of my name to the authorship list** /and the proposed change in corresponding author		

Both added/removed authors should complete the information in the first table under Section 6.

---- End of form ----
