# Supplementary material for: Management of acute mastoiditis in children: a retrospective analysis
Source: Ital J Pediatr. 2025 Sep 24;51:272. doi: 10.1186/s13052-025-02075-8 (PMC12462347; doi:10.1186/s13052-025-02075-8)
Supplement: Supplementary file 2 — Supplementary Material 2 [file 13052_2025_2075_MOESM2_ESM.doc]

Florence, 10^th^ June 2025

Dear Reviewers,

we thank you for your valuable comments, which we carefully considered, and addressed in the revised manuscript. Details of the revisions and responses are described below. To assist the reviewers, we have attached both a clean version and a track-changes version of the revised manuscript.

We believe that the manuscript has been much improved as a result of the revisions.

Sincerely yours,

Carlotta Montagnani, MD

Infectious Disease Unit, Department of Health Sciences, University of Florence

viale Pieraccini, 24, I-50139 Florence, Italy.

phone: +39 055-5662399; +39 3381909494

fax: +39-055-5662830

e-mail: carlotta.montagnani@meyer.it

Reviewer comments:

Reviewer #1: This retrospective study provides a valuable contribution to the literature that aims to shed light on important gaps in the management of acute mastoiditis (AM) and complicated mastoiditis (CAM) in children. This study, conducted with 8 years of data at Meyer Children's Hospital, provides comparative data on diagnosis, treatment, and outcome. However, some aspects of methodological improvement are still needed.
1. Strengths of the Article
Comprehensive data: Extensive patient data collected over an 8-year period provided important conclusions from both epidemiological and clinical perspectives.
Comparative approach: Systematic comparison of AM and CAM patients in terms of clinical features, laboratory values, treatment method and outcomes is well planned.
Current importance: The increase in cases detected in the post-COVID-19 period gives the study additional epidemiological importance.
Statistical analysis: Inclusion of ROC analysis and regression model for CRP is appropriate to demonstrate the value of biomarkers.

2. Methodological Deficiencies
Patient selection criteria: Although the definition of "complicated" (CAM) is clear, it is stated that the classification was made retrospectively. In this case, the accuracy of imaging findings and the effect of deficiencies in the records should be discussed in more detail.

*We agree with the reviewer and we added a list of radiological findings in Methods section. See line 84-6. We also acknowledged among the study’s limitations that its retrospective nature may affect the accuracy of the diagnosis (line 260-2).*

Treatment algorithm is missing: The specific diagnosis-treatment algorithm applied in the hospital (if any) is not presented to the reader. This information would be important for comparison.

*We highlighted the lack of a diagnostic-therapeutic algorithm in Methods and Discussion sections (line 94 and 240-2).*

Antibiotic treatment: It is stated that 18 different treatment protocols were used, but it was not analyzed which antibiotics were ineffective or not preferred in the development of CAM. Despite this diversity, no meaningful conclusion was made.

*We added a discussion on this point* *(line 240-2, 240-8).*

3. General evaluation
Language and fluency: Although the article is generally understandable, there are repetitions and long sentences in some paragraphs. More concise and focused expression should be preferred, especially in the introduction and discussion sections.

*We thank the Reviewer for this helpful observation. In response, we have carefully revised the manuscript, with particular attention to the Background and Discussion sections (s*ee *the version with tracked changes). We have eliminated redundant phrases, shortened overly long sentences, and improved the overall clarity and conciseness of the text. We believe these changes have enhanced the fluency and readability of the manuscript.*

Relation to literature: Although the findings are occasionally compared with the literature, some references (e.g. the importance of Pseudomonas) are left at a speculative level. These points should be supported or limited more clearly.

*We thank the Reviewer for this helpful observation. We have added more detailed explanations as requested by the reviewer (line 229-35) and updated the reference list accordingly.*

4. Conclusion
The basic structure of the study is solid, the data is meaningful and the potential to contribute to the literature is high. It may be at an acceptable level after the corrections I mentioned above are made.


Reviewer #2: The study presents a retrospective analysis of cases of acute mastoiditis (AM) and complicated acute mastoiditis (CAM) admitted to the Meyer Pediatric Hospital in Florence.
The paper is overall well written, but it requires some clarifications.

Does the case series include any instances of cochlear implants complicated by mastoid empyema?

*Patients with cochlear implants were excluded from the studies, we added it to Methods section (line 79).*

It would be important to reinforce the idea that, although European guidelines recommend the use of amoxicillin alone—which is often ineffective due to the development of multiple resistances—it would be more appropriate to use a combination of amoxicillin and clavulanic acid as a first-line treatment.

*We thank the Reviewer for this observation*. *We added some consideration about antibiotics at home as suggested by the reviewer (line 222-228)*

Abstract, line 40: I assume that the increase in AM cases reported in 2023 can be attributed to the post-COVID-19 pandemic period, when social contact restrictions were lifted. Please clarify this point.

*We reformulated the phrase (line 40)*

Background, line 49: Acute mastoiditis is the most common complication of acute otitis media, but its incidence can be classified as a rare complication, although it is serious due to its potential consequences. Please clarify this concept more precisely.

*We thank the Reviewer for this observation*. *We have addressed this point in greater depth* *(line 50-5).*

Results, line 132: While acknowledging the hospital's policies of cost containment and reduced radiation exposure, a "cone beam" CT scan—characterized by its speed and low radiation dose—should always be performed in hospitalized children with a severe clinical picture suspected for mastoiditis. This allows for assessment of the pathological condition of the middle ear and mastoid, and to exclude rare diseases such as congenital cholesteatoma with an intact tympanic membrane.

*We thank the Reviewer for this valuable suggestion. We fully agree on the utility of cone beam CT in the evaluation of children with suspected severe mastoiditis, particularly given its advantages in terms of image quality and reduced radiation exposure. However, we would like to clarify that cone beam CT is not currently available at our institution, and therefore was not utilized in the present study.*
